# Supplementary material for: Fecal Microbial Composition and Predicted Functional Profile in Irritable Bowel Syndrome Differ between Subtypes and Geographical Locations
Source: Microorganisms. 2023 Oct 5;11(10):2493. doi: 10.3390/microorganisms11102493 (PMC10608977; doi:10.3390/microorganisms11102493)
Supplement: Supplementary file 1 [file microorganisms-11-02493-s001.zip › microorganisms-2588610-Supplementary Information.pdf]

Fecal Microbial Composition and Predicted Functional Profile in Irritable Bowel Syndrome Differ Between Subtypes and Geographical Locations

(SUPPLEMENTARY INFORMATION)

Jose F. Garcia-Mazcorro [1], Mercedes Amieva-Balmori [2], Arturo Triana-Romero [2], Bridgette Wilson [3], Leanne Smith [3], Job Reyes-Huerta [2], Megan Rossi [2], Kevin Whelan [3] and Jose M. Remes-Troche [2,*]

[1] Research and Development, MNA de Mexico, San Nicolas de los Garza 66477, Mexico

[2] Instituto de Investigaciones Médico Biológicas, Universidad Veracruzana, Mexico

[3] Department of Nutritional Sciences, King's College London, United Kingdom

* Correspondence: jose.remes.troche@gmail.com

Summary. The aim of this study was to characterize fecal microbiota in patients with diagnosed IBS based on Rome IV criteria. This document contains supplementary information that supports the main document.

1. Inclusion and exclusion criteria

Inclusion criteria included interest in taking part, ability to give informed consent, men and women aged 18-65 years with diarrhea-predominant IBS (IBS-D), constipation-dominant IBS (IBS-C), or mixed (IBS-M), based on fulfilment of the Rome IV criteria for irritable bowel syndrome who do not have a major medical condition (e.g. diabetes, psychiatric or current eating disorders), severe oesophagitis, gastritis or duodenitis, gastrointestinal disease (inflammatory bowel disease, coeliac disease, active diverticulitis), or history of previous GI surgery (excluding appendicectomy, cholecystectomy and haemorrhoidectomy), severe renal, cardiac, pulmonary, or other chronic diseases likely to affect motility, history of gastric bezoars. Exclusion criteria included females who report to be pregnant or lactating, Body Mass Index (BMI) >40 kg/m2, use of unpermitted medications in the last 4 weeks prior to, or during the study, including antibiotics within the last 4 weeks, dietary fibre food supplements within the last 4 weeks (e.g. Fybogel, Lactulose), prebiotics or probiotics (in food products or as supplements) within the last 4 weeks, other dietary supplements that may affect the luminal microenvironment of the intestine (e.g. Orlistat), use of drugs known to alter GI motility, transit or gastric pH (e.g. mebeverine, opiates, monoamine oxidase inhibitors, phenothiazines) in the last 1 week, full bowel preparation for a diagnostic procedure within the last 4 weeks, changes to IBS medications or dose in the 4 weeks prior to the study, changes to anti-depressant medications or dose in the 12 weeks prior to the study, swallowing disorders (physical or psychological), use of implantable and/or medical devices such as pacemakers, individuals following extreme diets e.g. 8 or more caffeinated serves per day, 4 or more bottles of wine (40 or more units of alcohol per week) or equivalent per week as assessed by diet questionnaires or changes to smoking habits, individuals who have participated in

other intervention trials within 3 months prior to screening, allergies to components (soy) of the SmartBar (required for SmartPill protocol), abdominal pain for less than 2 days in the screening week (based on the GSRS mild to severe), those who report adequate relief of symptoms at baseline using the Global Symptom Questionnaire (GSQ). Participants were required to provide a stool sample using a kit provided by our research group. Stool samples were temporarily stored in the patients' own freezer and delivered within 1 hour after defecation.

2. DNA extraction and sequencing

Fecal samples from patients in Mexico were shipped at -20°C using an international carrier (https://www.marken.com/) on January 20th, 2020, and arrived in London 79
on January 24th, 2020, with no temperature deviations. Aliquots of 250 mg of stool were used for DNA extraction using the PowerLyzer PowerSoil DNA Isolation Kit (Qiagen, see: https://www.qiagen.com/us/products/discovery-and-translational-research/dna-rna-purification/dna-purification/microbial-dna/dneasy-powerlyzer-powersoil-kit/) at the Royal College of London. This kit includes a step of bead beating with 0.1 mm glass beads and a membrane for DNA purification. A semi-conserved region of the 16S rRNA gene was amplified using PCR with the following primers (515F: GTGYCAGCMGCCGCGGTAA, 806R: GGACTACNVGGGTWTCTAAT). PCR and 16S sequencing were performed at the Molecular Research LP (Shallowater, Texas, USA) in a MiSeq instrument (Illumina) following the manufacturer's instructions.

3. Statistical analyses

The use of relative abundance (i.e. percentage of 16S sequences) has historically been the data of choice to perform comparisons of microbial taxa in studies of gut microbial ecology. However, it is well known that relative abundance can lead to spurious correlations, originally pointed out by Karl Pearson more than a century ago. As a result, as some taxa increase in abundance others must decrease, thus leading to graphical presentations that do not reflect the true underlying relationships. We performed a series of experiments to test the performance of centered log-ratio (clr) transformation (Moossavi et al. 2019) and applied this transformation to the raw number of sequences obtained from the filtered table (features that were present with a frequency of <20 and presence in <10 samples, Estaki et al. 2020). Transformations were performed at each phylogenetic level separately. More details about this procedure, including its usefulness in studies of gut microbial ecology, have recently been published (Manzanares-Miranda et al. 2023).

4. Analysis of clr-transformed data at the phylum level

We used the following code in SAS University Edition to graphically explore the potential effect of age and BMI as continuous explanatory variables to explain possible differences in clr-transformed data at the phylum level between genders and countries. As an example, Figures S1 and S2 show regression plots for all clr-transformed data (n=120) and Mexican samples only (n=82) for Bacteroidetes with both age and BMI as covariates.

SAS code for graphical exploration:

```
proc sgplot data=import;
reg x= Age y=Firmicutes /group=Country clm; run;
```

Patterns worth exploring (i.e. slopes well separated) were analyzed using the following code in SAS.

SAS code for covariate analyses:

```
proc mixed data=import method=reml;
class Country;
model Bacteroidetes= Age Country Age*Country/e3 ddfm=kr solution residual; run;
```

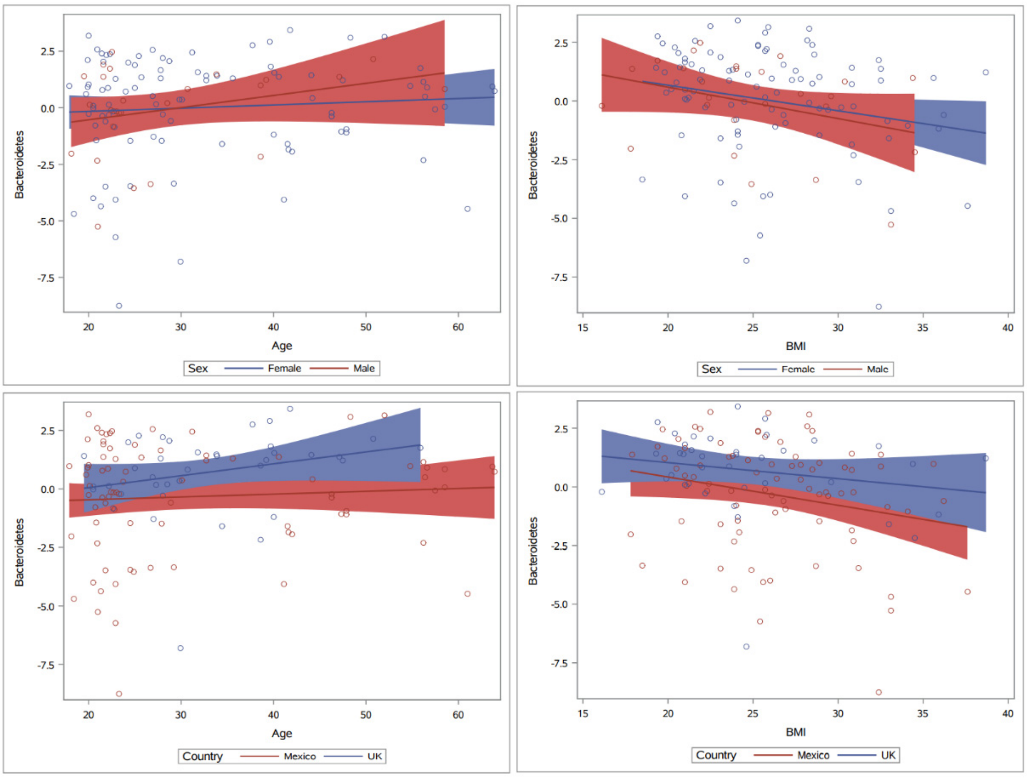

Figure S1. Regression plots for clr-transformed data for Bacteroidetes with 95% confidence limits for the mean. All samples were used (n=120) and organized according to Sex and Country. Age and BMI were used as potential explanatory variables. Plots were created using the SGPLOT procedure in SAS University Edition.

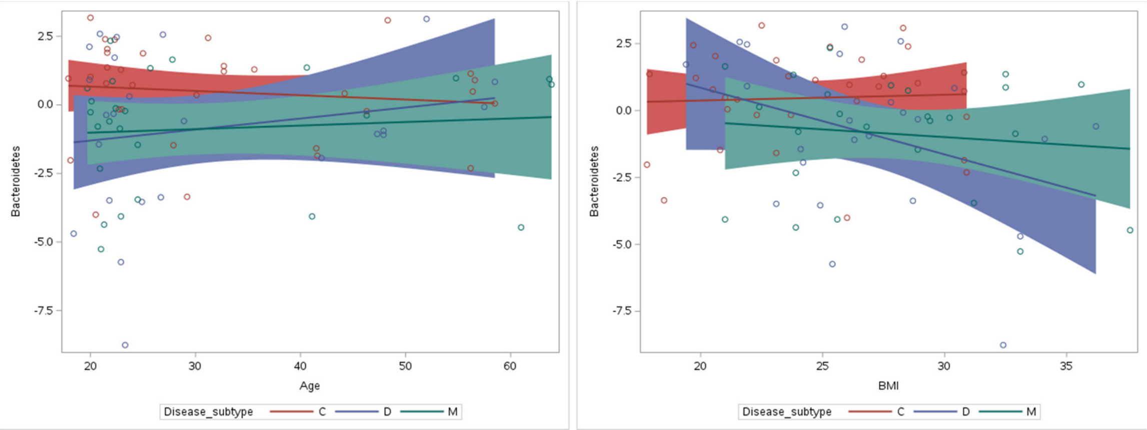

Figure S2. Regression plots for clr-transformed data for Bacteroidetes with 95% confidence limits for the mean. Samples from Mexican patients were used only (n=82) with age and BMI as potential explanatory variables. Plots were created using the SGPLOT procedure in SAS University Edition.

.

5. Analysis of clr-transformed data at the genus level

The following SAS codes were used for the analysis of clr-transformed data at the phylum and genus level, when appropriate. A summary of the statistical results from these analyses is shown in Table S1.

```
*For IBS subtype only:
proc mixed data=import method=reml;
class Disease_subtype;
model Bacteroides= Disease_subtype /e3 ddfm=kr solution residual;
run;
*For country only:
proc mixed data=import method=reml;
```

```
class Country;

model Bacteroides= Country /e3 ddfm=kr solution residual;

run;

*For sex only:

proc mixed data=import method=reml;

class Sex;

model Bacteroides= Sex /e3 ddfm=kr solution residual;

run;

*For interactions:

proc mixed data=import method=reml;

class Disease_subtype Country;

model Bacteroides= Disease_subtype Country Disease_subtype*Country/e3 ddfm=kr

solution residual;

run;
```

Table S1 Summary of statistical results at the genus level. Taxa that did not show any statistical significance (p>0.05) for both the analysis in PROC MIXED and Kruskal-Wallis (K-W) test are shown in gray. Taxa that showed consistent statistical significance (p<0.05) with both methods for at least one dependent variable (IBS subtype, country, or sex) are shown in blue.

**Taxon**	**IBS subtype**	**Country**	**Sex**
*Methanobrevibacter***	p=0.0597<br>C>D (p=0.0585)	p=0.6181	p=0.4161
Interactions		NS	NS
K-W test	p=0.0938	NS	NS
*Actinomyces***	p=0.9955	p=0.7123	p=0.5726
Interactions		NS	NS
K-W test	NS	NS	NS
*Varibaculum***	p=0.5716	p=0.0383<br>UK>Mexico (p=0.0383)	p=0.6940
Interactions		NS	NS
K-W test	NS	NS	NS
*Rothia***	p=0.8184	p=0.1451	p=0.4323
Interactions		NS	NS
K-W test	NS	NS	NS

*Bifidobacterium***	p=0.6082	p=0.2892	p=0.9049
Interactions		NS	NS
K-W test	NS	NS	NS
Coriobacteriaceae**	p=0.5901	p=0.4317	p=0.7736
Interactions		NS	NS
K-W test	NS	NS	NS
*Adlercreutzia***	p=0.6237	p=0.0270<br>UK>Mexico (p=0.0270)	p=0.7170
Interactions		NS	NS
K-W test	NS	p=0.0015	NS
*Collinsella***	p=0.5403	p=0.0608	p=0.1195
Interactions		NS	NS
K-W test	NS	p=0.0590	NS
*Eggerthella***	p=0.9683	p=0.3615	p=0.3663
Interactions		NS	NS
K-W test	NS	NS	NS
*Slackia***	p=0.0800	p=0.0116<br>Mexico>UK (p=0.0116)	p=0.8146
Interactions		Interaction: p=0.0050	NS
K-W test	NS	p=0.0122	NS
Bacteroidales (unassigned genus)**	p=0.2720	p=0.0688	p=0.7519
Interactions		NS	NS
K-W test	NS	NS	NS
*Bacteroides**	p=0.0373<br>C>M (p=0.0432)	p=0.0218<br>UK>Mexico (p=0.0218)	p=0.8388
Interactions		NS	NS
K-W test	p=0.0267	p=0.0104	NS
*Parabacteroides***	p=0.5920	p=0.0570<br>UK>Mexico (p=0.0570)	p=0.7577
Interactions		NS	NS
K-W test	NS	p=0.0460	NS
*Prevotella***	p=0.9242	p=0.1067	p=0.4920
Interactions		NS	NS
K-W test	NS	NS	NS
Rikenellaceae**	p=0.6218	p=0.0164<br>UK>Mexico (p=0.0164)	p=0.1820
Interactions		NS	NS
K-W test	NS	p=0.0298	NS
Rikenellaceae2**	p=0.0440<br>C>D (p=0.0570)	p=0.1400	p=0.1996
Interactions		NS	NS
K-W test	p=0.0521	p=0.0545	p=0.1307
*Alistipes***	p=0.3197	p=0.0186<br>UK>Mexico (p=0.0186)	p=0.3882
Interactions		NS	NS
K-W test	NS	p=0.0140	NS
Rikenellaceae3**	p=0.9281	p=0.0026<br>UK>Mexico (p=0.0026)	p=0.2891
Interactions		NS	NS
K-W test	NS	p=0.0029	NS
S24-7**	p=0.0818	p=0.2735	p=0.9256
Interactions		NS	NS
K-W test	NS	NS	NS

Barnesiellaceae**	p=0.2099	p=0.0143<br>UK>Mexico (p=0.0143)	p=0.5849
Interactions		NS	NS
K-W test	NS	p=0.0168	NS
*Butyricimonas***	p=0.0452<br>C>D (p=0.0370)	p=0.1286	p=0.3097
Interactions		NS	NS
K-W test	p=0.0559	NS	NS
CF231**	p=0.5123	p=0.1425	p=0.6010
Interactions		NS	NS
K-W test	NS	NS	NS
*Paraprevotella***	p=0.6230	p=0.0276<br>Mexico>UK (p=0.0276)	p=0.6050
Interactions		NS	NS
K-W test	NS	p=0.0301	NS
*Prevotella*2**	p=0.6158	p=0.6121	p=0.6302
Interactions		NS	NS
K-W test	NS	NS	NS
YS2 (Cyanobacteria)**	p=0.1465	p=0.0290<br>UK>Mexico (p=0.0290)	p=0.2528
Interactions		NS	NS
K-W test	NS	p=0.0536	NS
*Streptophyta***	p=0.2674	p=0.9569	p=0.2729
Interactions		NS	NS
K-W test	NS	NS	NS
*Staphylococcus***	p=0.0072<br>M>C (p=0.0080)	p=0.0054<br>Mexico>UK (p=0.0054)	p=0.2841
Interactions		NS	NS
K-W test	p=0.0142	p=0.0051	NS
Lactobacillales (unassigned genus)**	p=0.1018	p=0.0002<br>Mexico>UK (p=0.0002)	p=0.9672
Interactions		NS	
K-W test	NS	p=0.0004	NS
*Enterococcus***	p=0.0026<br>M>D (p=0.0021)	p<0.0001<br>Mexico>UK (p<0.001)	p=0.1467
Interactions		NS	NS
K-W test	p=0.0040	p<0.0001	NS
*Vagococcus***	p=0.0438<br>M>C (p=0.0483)	p=0.0067<br>Mexico>UK (p=0.0067)	p=0.2006
Interactions		NS	p=0.0017
K-W test	NS	p=0.0051	NS
Lactobacillaceae**	p=0.5664	p=0.1064	p=0.5960
Interactions		NS	NS
K-W test	NS	NS	NS
*Lactobacillus***	p=0.7568	p=0.0945	p=0.1480
Interactions		p=0.0652	p=0.3291
K-W test	NS	NS	NS
*Pediococcus***	p=0.0696	p=0.4621	p=0.6239
Interactions		NS	NS
K-W test	p=0.0293	NS	NS
*Leuconostoc***	p=0.1016	p=0.0074<br>Mexico>UK (p=0.0074)	p=0.8149
Interactions		NS	NS
K-W test	NS	p=0.0107	NS

*Weissella***	p=0.1867	p=0.0090	p=0.1249
Interactions		NS	NS
K-W test	NS	p=0.0037	NS
*Lactococcus***	p=0.0186<br>M>D (p=0.0177)	p<0.0001<br>Mexico>UK (p<0.0001)	p=0.5571
Interactions		NS	NS
K-W test	p=0.0142	p<0.0001	NS
*Streptococcus**	p=0.9974	p=0.0017<br>Mexico>UK (p=0.0017)	p=0.3437
Interactions		p=0.0116	NS
K-W test	NS	p=0.0018	NS
*Turicibacter***	p=0.5977	p=0.9731	p=0.4236
Interactions		NS	NS
K-W test	NS	NS	NS
Clostridiales (unassigned genus)*	p=0.1414	p=0.4286	p=0.3107
Interactions		NS	NS
K-W test	NS	NS	NS
Clostridiales2*	p=0.7091	p=0.6859	p=0.1978
Interactions		p=0.0133	NS
K-W test	NS	NS	NS
Christensenellaceae**	p=0.0486<br>C>D (p=0.0467)	p=0.7398	p=0.1362
Interactions		NS	NS
K-W test	NS	NS	NS
Christensenella**	p=0.0067<br>C>D (p=0.0049)	p=0.0888	p=0.0575<br>Females>Males<br>(p=0.0575)
Interactions		p=0.0499	p=0.0728
K-W test	p=0.0103	NS	p=0.0692
Clostridiaceae**	p=0.3066	p=0.0086<br>Mexico>UK (p=0.0086)	p=0.1909
Interactions		p=0.0416	NS
K-W test	NS	p=0.0040	NS
G02d06 (family Clostridiaceae)**	p=0.0165<br>C>D (p=0.0118)	p=0.1776	p=0.2311
Interactions		NS	NS
K-W test	P=0.0251	NS	NS
*Clostridium**	p=0.6794	p=0.7980	p=0.8192
Interactions		NS	NS
K-W test	NS	NS	NS
*Sarcina***	p=0.6467	p=0.1958	p=0.3183
Interactions		NS	NS
K-W test	NS	NS	NS
*Dehalobacterium***	p=0.2149	p=0.1136	p=0.0436<br>Females>Males<br>(p=0.0436)
Interactions		NS	NS
K-W test	NS	NS	p=0.0371
*Anaerofustis***	p=0.8591	p=0.0201<br>UK>Mexico (p=0.0201)	p=0.3665
Interactions		NS	NS
K-W test	NS	p=0.0178	NS
*Pseudoramibacter***	p=0.0168	p=0.0565	p=0.1271

	C>D (p=0.0128)	Mexico>UK (p=0.0565)	
Interactions		NS	NS
K-W test	p=0.0454	p=0.0369	NS
Lachnospiraceae*	p=0.2969	p=0.3835	p=0.6573
Interactions		NS	NS
K-W test	NS	NS	NS
Lachnospiraceae2*	p=0.1799	p=0.0850	p=0.1318
Interactions		NS	NS
K-W test	NS	NS	NS
*Anaerostipes***	p=0.5133	p=0.1013	p=0.7911
Interactions		NS	NS
K-W test	NS	NS	NS
*Blautia**	p=0.7058	p=0.6010	p=0.1561
Interactions		NS	NS
K-W test	NS	NS	NS
*Butyrivibrio***	p=0.5016	p=0.2257	p=1
Interactions		NS	NS
K-W test	NS	NS	NS
*Clostridium*2**	p=0.6764	p=0.1662	p=0.4448
Interactions		p=0.0431	p=0.2125
K-W test	NS	NS	NS
*Coprococcus**	p=0.4049	p=0.0091<br>UK>Mexico (p=0.0091)	p=0.6016
Interactions		p=0.0317	NS
K-W test	NS	p=0.0095	NS
*Dorea**	p=0.9032	p=0.0590<br>Mexico>UK (p=0.0590)	p=0.9430
Interactions		p=0.0299	p=0.5049
K-W test	NS	p=0.0516	NS
Lachnobacterium**	p=0.4215	p=0.5389	p=0.8253
Interactions		p=0.0052	p=0.0702
K-W test	NS	NS	NS
*Lachnospira***	p=0.7610	p=0.8847	p=0.1666
Interactions		NS	NS
K-W test	NS	NS	NS
*Lactonifactor***	p=0.3951	p=0.3746	p=0.8661
Interactions		NS	NS
K-W test	NS	NS	NS
*Roseburia***	p=0.7714	p=0.8357	p=0.7289
Interactions		NS	NS
K-W test	NS	NS	NS
Ruminococcus (Lachnospiraceae)**	p=0.7968	p=0.8009	p=0.1674
Interactions		NS	NS
K-W test	NS	NS	NS
*Shuttleworthia***	p=0.5333	p=0.0175<br>UK>Mexico (p=0.0175)	p=0.7572
Interactions		p=0.0674	NS
K-W test	NS	p=0.0103	NS
Ruminococcus (Lachnospiraceae 2)*	p=0.4042	p=0.8803	p=0.2231
Interactions		NS	NS
K-W test	NS	NS	NS
*Peptococcus***	p=0.8320	p=0.0134	p=0.2821

		Mexico>UK (p=0.0134)	
Interactions		NS	NS
K-W test	NS	p=0.0098	NS
Peptostreptococcaceae	p=0.7483	p=0.5951	p=0.7346
Interactions		NS	NS
K-W test	NS	NS	NS
Peptostreptococcaceae2**	p=0.8548	p=0.8722	p=0.1847
Interactions		NS	NS
K-W test	NS	NS	NS
Clostridium 3	p=0.6487	p=0.0656	p=0.5620
Interactions		p=0.0134	NS
K-W test	NS	NS	NS
Ruminococcaceae (unassigned genus)*	p=0.0782	p=0.2377	p=0.8464
Interactions		NS	NS
K-W test	p=0.0603	NS	NS
Ruminococcaceae (unassigned genus 2)*	p=0.1984	p=0.8317	p=0.0134 Females>Males (p=0.0134)
Interactions		NS	NS
K-W test	NS	NS	NS
*Anaerotruncus***	p=0.0708	p=0.2988	p=0.2252
Interactions		p=0.0723	NS
K-W test	p=0.0699	NS	NS
*Butyricicoccus***	p=0.2813	p=0.5111	p=0.4940
Interactions		NS	NS
K-W test	NS	NS	NS
*Faecalibacterium**	p=0.0020 D>M (p=0.0013)	p=0.0005 UK>Mexico (p=0.0005)	p=0.0650
Interactions		NS	NS
K-W test	p=0.0007	p<0.0001	p=0.0318
*Gemmiger**	p=0.9703	p=0.0211 UK>Mexico (p=0.0211)	p=0.5567
Interactions		NS	NS
K-W test	NS	p=0.0199	NS
*Oscillospira**	p=0.1148	p=0.5969	p=0.3897
Interactions		NS	NS
K-W test	NS	NS	NS
Ruminococcus (Ruminococcaceae)*	p=0.5226	p=0.0889	p=0.3207
Interactions		NS	p=0.0251
K-W test	NS	NS	NS
*Acidaminococcus***	p=0.4666	p=0.6014	p=0.9506
Interactions		NS	NS
K-W test	NS	NS	NS
*Dialister**	p=0.0778	p=0.0001 Mexico>UK (p=0.0001)	p=0.5956
Interactions		NS	NS
K-W test	NS	p=0.0002	NS
*Megamonas***	p=0.3348	p=0.8783	p=0.3710
Interactions		NS	NS
K-W test	NS	NS	NS
*Megasphaera***	p=0.9497	p=0.0005 Mexico>UK (p=0.0005)	p=0.9793

Interactions		NS	NS
K-W test	NS	p=0.0010	NS
*Mitsuokella***	p=0.6527	p=0.1553	p=0.5992
Interactions		NS	p=0.0006
K-W test	NS	NS	NS
*Phascolarctobacterium***	p=0.0080<br>C>D (p=0.0142)<br>C>M (p=0.0266)	p=0.9717	p=0.9101
Interactions		p=0.0517	NS
K-W test	p=0.0137	NS	NS
*Succiniclasticum***	p=0.6353	p=0.0615	p=0.5060
Interactions		NS	NS
K-W test	NS	p=0.0370	NS
*Veillonella***	p=0.0998	p=0.9020	p=0.2833
Interactions		NS	NS
K-W test	NS	NS	NS
Mogibacteriaceae*	p=0.6578	p=0.0901	p=0.1671
Interactions		NS	NS
K-W test	NS	NS	NS
*Mogibacterium***	p=0.0616	p=0.0077<br>Mexico>UK (p=0.0077)	p=0.4094
Interactions		NS	NS
K-W test	NS	p=0.0122	NS
*Parvimonas***	p=0.7041	p=0.5209	p=0.7494
Interactions		NS	NS
K-W test	NS	NS	NS
Erysipelotrichaceae**	p=0.4058	p=0.0072<br>UK>Mexico (p=0.0072)	p=0.1827
Interactions		NS	p=0.0374
K-W test	NS	p=0.0066	NS
*Bulleidia***	p=0.3758	p=0.1605	p=0.8304
Interactions		NS	NS
K-W test	NS	NS	NS
*Catenibacterium***	p=0.3001	p=0.0009<br>Mexico>UK (p=0.0009)	p=0.8390
Interactions			p=0.0255
K-W test	NS	p=0.0007	NS
Clostridium (family Erysipelotrichaceae)**	p=0.0129<br>D>C (p=0.0100)	p=0.1358	p=0.0901
Interactions		NS	NS
K-W test	p=0.0170	NS	NS
*Coprobacillus***	p=0.5938	p=0.3826	p=0.0353<br>Females>Males<br>(p=0.0353)
		NS	NS
	NS	NS	p=0.0328
*Eubacterium**	p=0.6352	p=0.0051<br>Mexico>UK (p=0.0051)	p=0.3258
Interactions		NS	NS
K-W test	NS	p=0.0083	NS
cc-115 (family Erysipelotrichaceae)**	p=0.4423	p=0.0932	p=0.4888
Interactions		p=0.0900	p=0.5884
K-W test	NS	p=0.0623	NS

Fusobacteriaceae**	p=0.6762	p=0.1895	p=0.0036<br>Males>Females (p=0.0036)
Interactions		NS	p=0.0808
K-W test	NS	NS	p=0.0080
Fusobacteriaceae2**	p=0.8123	p=0.2748	p=0.0188<br>Males>Females (p=0.0188)
		NS	NS
	NS	NS	p=0.0738
*Fusobacterium***	p=0.1358	p=0.1830	p=0.8209
		NS	NS
	NS	NS	NS
Victivallaceae**	p=0.1340	p=0.1258	p=0.9286
		NS	NS
	NS	NS	NS
Alphaproteobacteria**	p=0.0429<br>C>D (0.0555)	p=0.2397	p=0.3643
Interactions		NS	NS
K-W test	p=0.0421	NS	NS
RF32**	p=0.5187	p=0.0229<br>UK>Mexico (p=0.0229)	p=0.5548
Interactions		NS	NS
K-W test	NS	p=0.0169	NS
Sutterella	p=0.9569	p=0.3738	p=0.9178
Interactions		NS	NS
K-W test	NS	NS	NS
*Bilophila***	p=0.0083<br>D>M (p=0.0083)	p<0.0001<br>UK>Mexico (p<0.0001)	p=0.3589
Interactions		NS	NS
K-W test	p=0.0070	p<0.0001	NS
*Desulfovibrio***	p=0.3503	p=0.0080<br>Mexico>UK (p=0.0080)	p=0.8170
Interactions		NS	NS
K-W test	NS	p=0.0035	NS
Enterobacteriaceae (unassigned genus)*	p=0.0095<br>C>D (p=0.0468)<br>M>D (p=0.0174)	p<0.0001<br>Mexico>UK (p<0.0001)	p=0.0879
Interactions		NS	NS
K-W test	p=0.0206	<0.0001	p=0.1112
*Serratia***	p=0.1644	p=0.6993	p=0.8186
Interactions		NS	NS
K-W test	NS	NS	NS
*Shigella***	p=0.4127	p=0.0253<br>Mexico>UK (p=0.0253)	p=0.3274
Interactions		NS	NS
K-W test		p=0.0359	
*Synergistes***	p=0.0002<br>C>D (p=0.0005)<br>C>M (p=0.0013)	p=0.0314<br>Mexico>UK (p=0.0314)	p=0.8151
Interactions		NS	NS
K-W test	p=0.0002	p=0.0253	NS
RF-39 (Tenericutes)**	p=0.2360	p=0.0087<br>Mexico>UK (p=0.0087)	p=0.5446

Interactions		NS	NS
K-W test	NS	p=0.0120	NS
ML615J28**	p=0.6200	p=0.9305	p=0.7982
Interactions		NS	NS
K-W test	NS	NS	NS
*Akkermansia***	p=0.729	p=0.4159	p=0.0915
Interactions		NS	NS
K-W test	NS	NS	NS
**Odd residuals that did not improve with random effects alone or nested (e.g. within gender or country). *Normally distributed residuals. NS: not significant.			

6. Alpha diversity analyses

We used the following code in SAS to graphically explore the potential effect of age and BMI as continuous explanatory variables to explain possible differences in alpha diversity metrics between genders and countries. Figures S3 to S6 show regression plots with age and BMI as continuous variables for samples divided by both country and sex.

```
proc sgplot data=import;
reg x= Age y=ASVs /group=Country clm;
run;
```

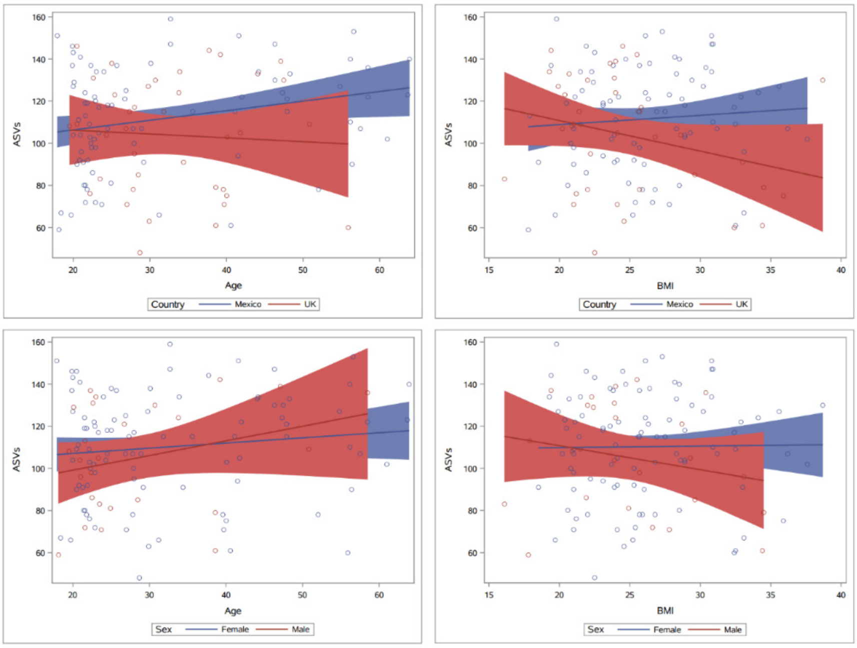

Figure S3. Regression plot for ASVs with 95% confidence limits for the mean. Plots were created using the SGPLOT procedure in SAS University Edition.

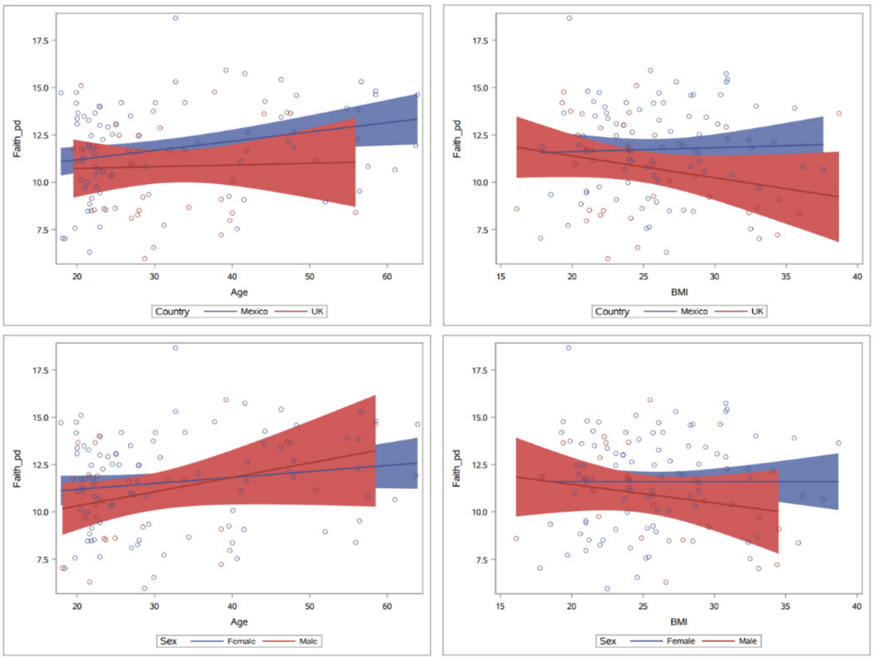

Figure S4. Regression plot for Faith_pd with 95% confidence limits for the mean. Plots were created using the SGPLOT procedure in SAS University Edition.

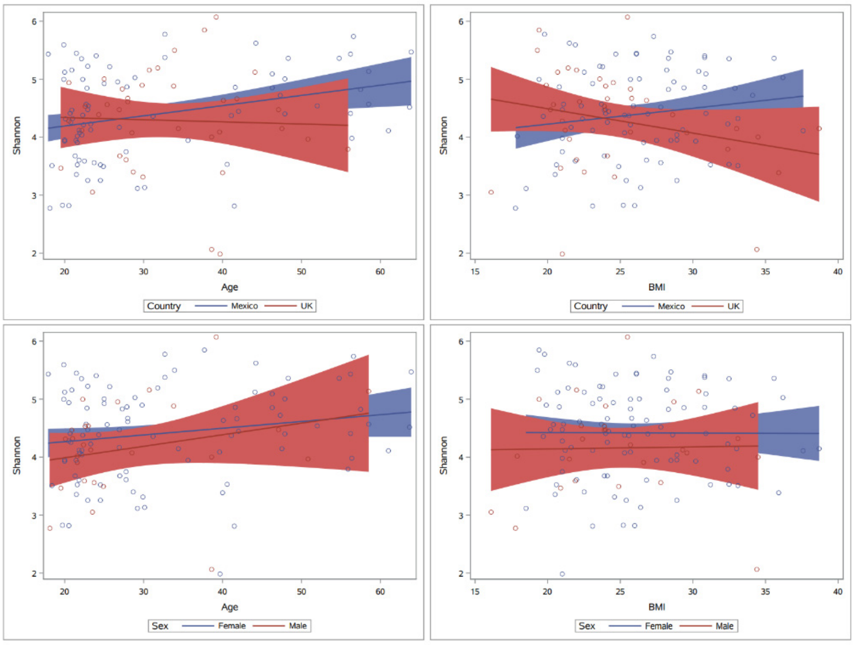

Figure S5. Regression plot for Shannon indexes with 95% confidence limits for the mean. Plots were created using the SGPLOT procedure in SAS University Edition.

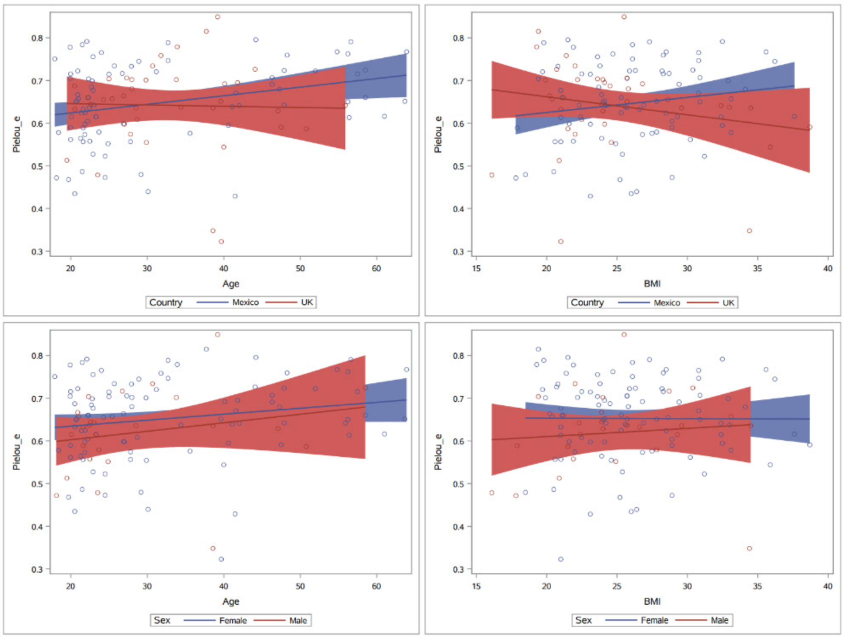

Figure S6. Regression plot for Pielou evenness with 95% confidence limits for the mean. Plots were created using the SGPLOT procedure in SAS University Edition.

7. Prediction of functional potential

Table S2 contains information about the 20 most significant results obtained from PICRUSt2 results from the comparison of Mexico vs UK, and Table S3 contains information about the 20 most significant results obtained from PICRUSt2 results for the analysis of the variable Disease*Country with 6 levels.

Table S2 Summary of the 20* most significant features that were significantly different between Mexican and British patients.

Observation Ids	p-values (corrected)	Description
ORNARGDEG-PWY<br>Expected Taxonomic Range: Bacteria	7.45E-08<br>↑ Mexico	Superpathway of L-arginine and L-ornithine degradation. This superpathway integrates the conversion of the amino acids L-arginine and L-ornithine to the polyamine putrescine, its subsequent degradation to 4-aminobutanoate, and finally the conversion of 4-aminobutanoate to succinate, which is fed into the TCA cycle.
PWY0-1338<br>Expected Taxonomic Range: Proteobacteria	8.69E-08<br>↑ Mexico	Polymyxin resistance. Polymyxins are antibiotics with a general structure consisting of a cyclic peptide with a long hydrophobic tail. They are produced by the Gram-positive bacterium Paenibacillus polymyxa and are selectively toxic for Gram-negative bacteria due to their specificity for the lipopolysaccharide molecules that exist within many Gram-negative outer membranes.
PWY-7446<br>Expected Taxonomic Range: Proteobacteria	8.77E-08<br>↑ Mexico	Sulfoquinovose degradation I. Sulfoquinovose is a major component of organo-sulfur compounds in nature. It is synthesized by higher plants, mosses, ferns, algae and most photosynthetic bacteria and serves as the polar headgroup of the sulfolipid in photosynthetic membranes.
ARGDEG-PWY<br>Expected Taxonomic Range: Bacteria	9.31E-08<br>↑ Mexico	Superpathway of L-arginine, putrescine, and 4-aminobutanoate degradation. This superpathway integrates the conversion of the amino acid L-arginine to the polyamine putrescine, its subsequent degradation to 4-aminobutanoate, and finally the conversion of 4-aminobutanoate to succinate, which is fed into the TCA cycle.
PWY-5863<br>Expected Taxonomic Range: Cyanobacteria, Viridiplantae	1.59E-07<br>↑ Mexico	Superpathway of phylloquinol biosynthesis. Phylloquinone (vitamin K1, 2-methyl-3-phytyl-1,4-naphthoquinone) acts as the electron transfer cofactor A1 of photosystem I (PS I) in higher plants and cyanobacteria. In non-photosynthetic microbes the very similar compound a menaquinone plays an essential role in several anaerobic electron transport systems.
AEROBACTINSYN-PWY<br>Expected Taxonomic Range: Proteobacteria	1.63E-07<br>↑ Mexico	Aerobactin biosynthesis. Iron is an essential trace element. In the presence of oxygen, ferrous iron is oxidized to ferric iron which forms insoluble compounds, and is thus not available to organisms. As a result, the level of physiologically available iron can drop to far below 1 μM, and become growth-limiting for bacteria. To survive, many bacteria evolved specialized transport systems called siderophores, which can complex and retract ferric iron ions.
AST-PWY<br>Expected Taxonomic Range: Proteobacteria	1.66E-07<br>↑ Mexico	L-arginine degradation II (AST pathway). The arginine succinyltransferase (AST) pathway is a catabolic pathway whose purpose is the dissimilation of the carbon skeleton of L-arginine. The pathway was detected in many Gram-negative proteobacteria able to utilize arginine as a sole carbon and nitrogen source.
PWY-5898<br>Expected Taxonomic Range: Agromyces, Microbacterium, Prevotella	1.67E-07<br>↑ Mexico	Superpathway of menaquinol-12 biosynthesis. Menaquinones (MK) and demethylmenaquinones (DMK) are low-molecular weight lipophilic components of the cytoplasmic membrane, found in many bacterial species. These quinones function as a reversible redox component of the electron transfer chain, mediating electron transfer between hydrogenases and cytochromes. Menaquinones have also been implicated in regulation, as they are necessary for sporulation and proper regulation of cytochrome formation in some Gram-positive bacteria.
PWY-5837	1.74E-07<br>↑ Mexico	2-carboxy-1,4-naphthoquinol biosynthesis. 1,4-dihydroxy-2-naphthoate is a branch point metabolite leading to the biosynthesis of menaquinone

Expected Taxonomic Range: Bacteria, Viridiplantae		(vitamin K2, in bacteria), phylloquinone (vitamin K1 in plants), and many plant pigments, including some two-ring naphthoquinones (such as lawsone and juglone) and the three-ring anthraquinones (such as alizarin and munjistin).
KDO-NAGLIPASYN-PWY<br>Expected Taxonomic Range: Aquificae, Bacteroidetes/Chlorobi group, Caldiserica, Chloroflexi, Chrysiogenetes, Cyanobacteria, Deferribacteres, Deinococcus-Thermus, Dictyoglomi, Elusimicrobia, FCB group, Fusobacteria, Gemmatimonadetes, Nitrospirae, Proteobacteria, PVC group, Spirochaetes, Synergistetes, Tenericutes, Thermodesulfobacteria, Thermotogae	1.80E-07<br>↑ Mexico	Superpathway of (Kdo)2-lipid A biosynthesis. Lipid A is the hydrophobic anchor of the outer membrane lipopolysaccharide of Gram-negative bacteria . Free lipid A does not exist as such in cells. It is normally found glycosylated with two KDO (3-deoxy-D-manno-octulosonic acid) residues and acylated with laurate and myristate residues.
PWY-5899<br>Expected Taxonomic Range: Microbacterium, Prevotella	1.85E-07<br>↑ Mexico	Superpathway of menaquinol-13 biosynthesis. Menaquinones (MK) and demethylmenaquinones (DMK) are low-molecular weight lipophilic components of the cytoplasmic membrane, found in many bacterial species. These quinones function as a reversible redox component of the electron transfer chain, mediating electron transfer between hydrogenases and cytochromes. Menaquinones have also been implicated in regulation, as they are necessary for sporulation and proper regulation of cytochrome formation in some Gram-positive bacteria.
PWY-5840<br>Expected Taxonomic Range: Archaea, Bacteria	2.08E-07<br>↑ Mexico	Superpathway of menaquinol-7 biosynthesis. Menaquinones (MK) and demethylmenaquinones (DMK) are low-molecular weight lipophilic components of the cytoplasmic membrane, found in many bacterial species. These quinones function as a reversible redox component of the electron transfer chain, mediating electron transfer between hydrogenases and cytochromes. Menaquinones have also been implicated in regulation, as they are necessary for sporulation and proper regulation of cytochrome formation in some Gram-positive bacteria
PWY-5897<br>Expected Taxonomic Range: Bacteroides, Micrococcales, Phocaeicola, Prevotella	2.09E-07<br>↑ Mexico	Superpathway of menaquinol-11 biosynthesis. Menaquinones (MK) and demethylmenaquinones (DMK) are low-molecular weight lipophilic components of the cytoplasmic membrane, found in many bacterial species. These quinones function as a reversible redox component of the electron transfer chain, mediating electron transfer between hydrogenases and cytochromes. Menaquinones have also been implicated in regulation, as they are necessary for sporulation and proper regulation of cytochrome formation in some Gram-positive bacteria.
PWY-5838<br>Expected Taxonomic Range: Bacteria, Halobacteria	3.73E-07<br>↑ Mexico	Superpathway of menaquinol-8 biosynthesis I. Menaquinones (MK) and demethylmenaquinones (DMK) are low-molecular weight lipophilic components of the cytoplasmic membrane, found in many bacterial species. These quinones function as a reversible redox component of the electron transfer chain, mediating electron transfer between hydrogenases and cytochromes. Menaquinones have also been implicated in regulation, as they are necessary for sporulation and proper regulation of cytochrome formation in some Gram-positive bacteria.
PWY-5861<br>Expected Taxonomic Range: Bacteria	3.87E-07<br>↑ Mexico	Superpathway of demethylmenaquinol-8 biosynthesis I. Menaquinones (MK) and demethylmenaquinones (DMK) are low-molecular weight lipophilic components of the cytoplasmic membrane, found in many bacterial species. These quinones function as a reversible redox component of the electron transfer chain, mediating electron transfer between hydrogenases and cytochromes. Menaquinones have also been implicated in regulation, as they are necessary for sporulation and proper regulation of cytochrome formation in some Gram-positive bacteria
PWY0-321<br>Expected Taxonomic Range: Bacteria, Fungi	4.41E-07<br>↑ Mexico	Phenylacetate degradation I (aerobic). Phenylacetate is a major intermediate in bacterial degradation of many aromatic compounds. Microbes can oxidize phenylacetate under both aerobic and anaerobic conditions (see also phenylacetate degradation II (anaerobic)), and the first step in both pathways is the same - the activition of phenylacetate to phenylacetyl-CoA by a phenylacetate-CoA ligase.
PWY-6071<br>Expected Taxonomic Range: Bacteria	4.89E-07<br>↑ Mexico	Superpathway of phenylethylamine degradation. *Escherichia coli* K-12 is capable of growth on 2-phenylethylamine as the sole carbon and energy source. 2-phenylethylamine is degraded to phenylacetate in two steps, involving a copper-containing amine oxidase and phenylacetaldehyde dehydrogenase. phenylacetate is then degraded to succinyl-CoA, an intermediate of the TCA cycle I (prokaryotic).

PWY-6629<br>Expected Taxonomic Range: Bacteria	6.39E-07<br>↑ Mexico	Superpathway of L-tryptophan biosynthesis. The aromatic amino acid L-tryptophan is biosynthesized from the principal precursors D-erythrose 4-phosphate and phosphoenolpyruvate via chorismate, a common precursor of many aromatic compounds including L-tyrosine, L-phenylalanine, and several other essential compunds, as shown in the superpathway of chorismate metabolism.
THREOCAT-PWY<br>Expected Taxonomic Range: Bacteria	6.90E-07<br>↑ Mexico	Superpathway of L-threonine metabolism. L-threonine is an indispensable amino acid. It is degraded by a complex network of pathways and regulatory signals. Two of the major routes for the degradation of L-threonine are known to occur both in mammals and in microorganisms. In the first route threonine is catabolized by catabolic threonine dehydratase (EC 4.3.1.19) to ammonia and 2-oxobutanoate, which is rapidly and irreversibly converted to propanoyl-CoA and formate (see L-threonine degradation I). A biosynthetic version of this enzyme has also been reported in certain anaerobic bacteria.
PWY-5850<br>Expected Taxonomic Range: Bacteria	1.06E-06<br>↑ Mexico	Superpathway of menaquinol-6 biosynthesis. Menaquinones (MK) and demethylmenaquinones (DMK) are low-molecular weight lipophilic components of the cytoplasmic membrane, found in many bacterial species. These quinones function as a reversible redox component of the electron transfer chain, mediating electron transfer between hydrogenases and cytochromes. Menaquinones have also been implicated in regulation, as they are necessary for sporulation and proper regulation of cytochrome formation in some Gram-positive bacteria
Methanogenesis-PWY<br>Expected Taxonomic Range: Euryarchaeota	1.9E-06<br>↑ UK	Methanogenesis, the biological production of methane, is an anaerobic respiration process carried out by the methanogens, a group of microorganisms belonging to the Archaea domain. These organisms account for most of the biogenic methane production, which is estimated at 5x1014 g of methane per year.
*Here we also included the methanogenesis-PWY for the involvement of methane in IBS, particularly the constipation-predominant form (Ghoshal et al. 2016). The description of the pathways was obtained from the MetaCyc Metabolic Pathway Database (https://metacyc.org/).		

Table S3 Summary of the 20* most significant features that were significantly different when using the variable Disease*Country with 6 levels (Mexico and UK for all 3 IBS subtypes).

Observation Ids	p-values (corrected)	Description
PWY-5898	1.93E-05<br>↑ Mexico vs UK with IBS-D	See Table S2.
PWY-5899	2.12E-05<br>↑ Mexico vs UK with IBS-D	See Table S2.
ORNARGDEG-PWY	2.32E-05<br>↑ Mexico vs UK with IBS-D	See Table S2.
AST-PWY	2.32E-05<br>↑ Mexico vs UK with IBS-D	See Table S2.
PWY-5897	2.35E-05<br>↑ Mexico vs UK with IBS-D	See Table S2.
PWY-5840	2.41E-05<br>↑ Mexico vs UK with IBS-D	See Table S2.
PWY0-1338	2.55E-05<br>↑ Mexico vs UK with IBS-D	See Table S2.
PWY-5863	2.60E-05<br>↑ Mexico vs UK with IBS-D	See Table S2.
ARGDEG-PWY	2.89E-05<br>↑ Mexico vs UK with IBS-D	See Table S2.
PWY-5837	3.03E-05<br>↑ Mexico vs UK with IBS-D	See Table S2.
PWY-7446	3.14E-05<br>↑ Mexico vs UK with IBS-D	See Table S2.
KDO-NAGLIPASYN-PWY	5.55E-05<br>↑ Mexico vs UK with IBS-D	See Table S2.
PWY-5838	5.56E-05<br>↑ Mexico vs UK with IBS-D	See Table S2.
PWY-5861	5.66E-05	See Table S2.

	↑ Mexico vs UK with IBS-D	
AEROBACTINSYN-PWY	5.72E-05<br>↑ Mexico vs UK with IBS-D	See Table S2.
PWY0-321	1.02E-04<br>↑ Mexico vs UK with IBS-D	See Table S2.
THREOCAT-PWY	1.08E-04<br>↑ Mexico vs UK with IBS-D	See Table S2.
PWY-6071	1.16E-04<br>↑ Mexico vs UK with IBS-D	See Table S2.
PWY-6629	1.18E-04<br>↑ Mexico vs UK with IBS-D	See Table S2.
PWY-922	1.92E-04<br>↑ Mexico vs UK with IBS-D	Mevalonate pathway I (eukaryotes and bacteria). The biosynthesis of 3-methylbut-3-en-1-yl diphosphate (IPP) can occur via two distinct routes: the mevalonate pathway (MVA pathway, such as this pathway) and the methylerythritol phosphate pathway (MEP pathway). In the former, IPP is synthesized from the condensation of three acetyl-CoA molecules; in contrast, in the MEP pathway IPP is synthesized via the condensation of pyruvate and D-glyceraldehyde 3-phosphate.
Methanogenesis-PWY	0.0003<br>↑ UK vs Mexico with IBS-D	See Table S2.
*Here we also included the methanogenesis-PWY for the involvement of methane in IBS, particularly the constipation-predominant form (Ghoshal et al. 2016).		

References

Estaki, M.; Jiang, L.; Bokulich, N.A.; McDonald, D.; González, A.; Kosciolek, T.; Martino, C.; Zhu, Q.; Birmingham, A.; Vázquez-Baeza, Y.; et al. QIIME2 enables comprehensive end-to-end analysis of diverse microbiome data and comparative studies with publicly available data. *Curr. Protoc. Bioinformatics* **2020**, *70*, e100.

Ghoshal, U.; Shukla, R.; Srivastava, D.; Ghoshal, U.C. Irritable Bowel Syndrome, particularly the constipation-predominant form, involves an increase in *Methanobrevibacter smithii*, which is associated with higher methane production. *Gut Liver* **2016**, *10(6)*, 932-938.

Manzanares-Miranda, N.; Garcia-Mazcorro, J.F.; Pérez-Medina, E.B.; Vaquera-Vázquez, A.; Martínez-Ruiz, A.; Ramos-Zayas, Y.; Kawas, J.R. Microbial populations in ruminal liquid simples from young Beefmaster bulls at both extremes of RFI values. *Microorganisms* **2023**, *11*, 663.

Moossavi, S.; Sepehri, S.; Robertson, B.; Bode, L.; Goruk, S.; Field, C.J.; Lix, L.M.; de Souza, R.J.; Becker, A.B.; Mandhane, P.J.; et al. Composition and variation of the human milk microbiota are influenced by maternal and early-life factors. *Cell Host Microbe* **2019**, *25*, 324-335.
